# Multi-Delay Identification of Rare Earth Extraction Process Based on Improved Time-Correlation Analysis

**DOI:** 10.3390/s23031102

**Published:** 2023-01-18

**Authors:** Rongxiu Lu, Hongliang Liu, Hui Yang, Jianyong Zhu, Wenhao Dai

**Affiliations:** 1 School of Electrical and Automation Engineering, East China Jiaotong University, Nanchang 330013, China; 2 Key Laboratory of Advanced Control and Optimization of Jiangxi Province, Nanchang 330013, China

**Keywords:** rare earth extraction, time delay identification, grey correlation analysis, time-correlation, discrete state transition algorithm, wavelet neural network

## Abstract

The rare earth extraction process has significant time delay characteristics, making it challenging to identify the time delay and establish an accurate mathematical model. This paper proposes a multi-delay identification method based on improved time-correlation analysis. Firstly, the data are preprocessed by grey relational analysis, and the time delay sequence and time-correlation data matrix are constructed. The time-correlation analysis matrix is defined, and the $H_{\infty }$ norm quantifies the correlation degree of the data sequence. Thus the multi-delay identification problem is transformed into an integer optimization problem. Secondly, an improved discrete state transition algorithm is used for optimization to obtain multi-delay. Finally, based on an Neodymium (Nd) component content model constructed by a wavelet neural network, the performance of the proposed method is compared with the unimproved time delay identification method and the model without an identification method. The results show that the proposed algorithm improves optimization accuracy, convergence speed, and stability. The performance of the component content model after time delay identification is significantly improved using the proposed method, which verifies its effectiveness in the time delay identification of the rare earth extraction process.

## 1. Introduction

The rare earth extraction process includes dozens or even hundreds of extraction tanks. The mixing speed and time of each group of agitators are different, which affects the reaction and transmission time of materials, leading to multi-delay, so a large amount of data cannot be effectively utilized. Current modeling studies of the rare earth extraction process do not consider time delay or take it as a constant [1,2,3], resulting in a particular gap between the model and the extraction site. Therefore, it is significant to study how to identify multi-delay.

A series of solutions have been proposed for the problem of time delay identification. The step response method was used in the time delay identification of systems [4,5], but this method is susceptible to noise and requires a filter to remove high-frequency noise. Rad et al. [6] estimated the time delay of input and output signals based on the cross-correlation function, which cannot reflect their specific relationship, so the results are not ideal. Some scholars used a recursive least squares algorithm to identify the system with time delay [7,8,9], which is assumed to be known and may not be established in engineering practice. Neural networks were used to identify time delay, which have the problem of long training times and easily falling into local optima [10,11,12]. Liu et al. [13] developed a compressed sensing recovery algorithm for the multiple input single output finite impulse response systems with unknown time delay, but it was not easy to choose the optimal threshold. Chen et al. [14] proposed an effective identification model based on the Bayesian theorem for systems with unknown time delay. Wang et al. [15] proposed a parameter identification method of a fractional-order time delay system based on the Legendre wavelet, which reduces the effect of noise on the accuracy of parameter identification. Hofmann et al. [16] proposed an offline time-delay identification strategy based on falling film evaporator pilot plant experiments and obtained good results in both validation experiments, with and without evaporation. Prasad et al. [17] used fractional-order modeling technique to identify the parameters of Hammerstein structured nonlinear systems with discontinuous asymmetric (two segment piecewise-linear with a dead-zone) nonlinearity and input time delay. Li et al. [18] designed a discrete-time robust adaptive estimator to identify the time delay and sandwich system parameters. Meanwhile, they reconstructed the observation and augmented data to obtain the explicit expression of the delay parameter. To achieve an effective system control strategy and accurate response prediction, Liu et al. [19] proposed a new method to identify the parameters of linear time-delay differential systems by analyzing the frequency domain response of complex systems. To solve the influence of time delay on HVAC systems, Li et al. [20] introduced transfer entropy and proposed a model-free identification method based on the information theory framework. Ni et al. [21] studied the parameter estimation problem for a class of linear time-delay systems. Based on the frequency responses and harmonic balance methods and by means of the gradient search, a two-stage stochastic gradient and gradient-based iterative algorithm was developed by using the collected data under the sinusoidal excitation. The maximum likelihood method [22], variable structure observer [23], particle swarm optimization [24], and other methods have also been applied.

Industrial processes have become complex with the rapid development of science and technology, resulting in multi-delay, and the abovementioned methods have been unable to meet practical requirements. Xie et al. [25] improved the genetic algorithm, applied it to the identification of multi-delay, and obtained their optimal estimate, but the method requires an accurate system model. Huang et al. [26] proposed an improved cross-correlation function method and realized multi-delay identification of the alumina carbon separation process. Wang et al. [27] proposed a trend similarity analysis method and realized the multi-delay identification of the hydrocracking process. All the abovementioned methods have the problems of high computational redundancy and time consumption.

This paper draws on the successful application of the time-correlation analysis method [28] in the alumina carbon separation and evaporation processes. We propose an improved time-correlation analysis method for the rare earth extraction process. Based on field data and the improved method, the multi-delay identification problem is transformed into an unconstrained integer optimization problem without changing the time delay relationship. Since the discrete state transition algorithm [29] can effectively solve the unconstrained integer optimization problem [30], we adopt the improved algorithm to solve it. Experimental comparison and analysis show that the improved time-correlation analysis method has high speed, high accuracy, and good stability, and is suitable for the multi-delay identification of the rare earth extraction process.

## 2. Improvement of Time-Correlation Analysis Method

Time-correlation analysis is a time delay identification method based on the relationship between data sequences, which has the advantage of high efficiency. This paper improves its shortcomings in data preprocessing and selection.

### 2.1. Grey Relational Analysis

Grey relational analysis (GRA) is derived from the grey system theory in system science [31]. Its basic idea is to judge the tightness of sequence connections according to the similarity between the geometric shapes of sequence curves. The closer the curves, the more significant the correlation between sequences.

Compared with traditional multi-factor analysis methods (such as canonical correlation analysis and multiple linear regression), this method has lower data requirements and less computational burden. It is suitable for quantitative analysis of the dynamic development process and hence can be used to analyze the correlation between a large amount of process data obtained from a production site and the content of rare earth elements.

Let the time base of a critical process variable in *N* work units be $d=[d_{1},d_{2},\ldots ,d_{i},\ldots ,d_{N}]$, where i=1,2,…,N, $d_{i}=\tau_{i}/T$. *T* is the sampling period, the delay sequence is $\Gamma =[\tau_{1},\tau_{2},\ldots ,\tau_{i},\ldots ,\tau_{N}]$, and $\tau_{i}$ is the time delay of the *i*th work unit. The content of *n* rare earth elements and *m* process variables is obtained by *k* times sampling. The element component content $U_{j}^{\prime }\left(t\right)=\left[u_{1}^{\prime }\left(t\right),u_{2}^{\prime }\left(t\right),\ldots ,u_{j}^{\prime }\left(t\right),\ldots ,u_{n}^{\prime }\left(t\right)\right]$ are used as the reference sequence in the correlation analysis, where 1≤j≤n, 1≤t≤k, and $u_{j}^{\prime }\left(t\right)$ represents the *j*th rare earth element component content. The process variable data $E_{l}^{\prime }\left(t\right)=\left[e_{1}^{\prime }\left(t\right),e_{2}^{\prime }\left(t\right),\ldots ,e_{l}^{\prime }\left(t\right),\ldots ,e_{m}^{\prime }\left(t\right)\right]$ are used as the comparison sequence, where 1≤l≤m, 1≤t≤k, and $e_{l}^{\prime }\left(t\right)$ represents the *l*th process variable data.

The original data are normalized to eliminate the influence of different dimensions on the results. Standard processing methods include initialization and averaging. This paper adopts averaging,

(1) $$\left\{\begin{array}{c} U_{j}\left(t\right)=U_{j}^{\prime }\left(t\right)/\frac{1}{k}\sum_{t=1}^{k}U_{j}^{\prime }\left(t\right)\\ E_{l}\left(t\right)=E_{l}^{\prime }\left(t\right)/\frac{1}{k}\sum_{t=1}^{k}E_{l}^{\prime }\left(t\right) \end{array}\right.$$

where $U_{j}\left(t\right)$ is the processed reference sequence data, and $E_{l}\left(t\right)$ is the processed comparison sequence data.

The correlation coefficient is used to express the degree of closeness between the index values of the comparison and reference sequence in grey relational analysis. The higher the value, the greater the degree of proximity,

(2) $$\xi_{lj}\left(t\right)=\frac{\underset{l}{min}\underset{j}{min}|U_{j}\left(t\right)-E_{l}\left(t\right)|+\rho \underset{l}{max}\underset{j}{max}\left|U_{j}\left(t\right)-E_{l}\left(t\right)\right|}{|U_{j}\left(t\right)-E_{l}\left(t\right)|+\rho \underset{l}{max}\underset{j}{max}\left|U_{j}\left(t\right)-E_{l}\left(t\right)\right|}$$

where $\xi_{lj}\left(t\right)$ is the correlation coefficient of the *l*th characteristic variable corresponding to the content of the *j*th rare earth element component, and ρ∈[0,1] is the resolution coefficient, which we take as 0.5.

According to the correlation coefficient, the correlation degree between each process variable and the content of rare earth elements can be obtained as

(3) $$r_{lj}\left(t\right)=\frac{1}{k}\sum_{t=1}^{k}\xi_{lj}\left(t\right)$$

The correlation degree is sorted from large to small. If $r_{11}<r_{21}$, then the correlation degree between the comparison sequence $e_{2}\left(t\right)$ and the content of the first rare earth element component is greater than that of comparison sequence $e_{1}\left(t\right)$.

### 2.2. Time Delay Identification Method Based on Time-Correlation Analysis

The process variable with the highest gray correlation is taken as the key process variable, and its standardized data $e_{l}\left(t\right)$ are used to form the time-correlation data matrix,

(4) $$E=\left|\begin{array}{cccccc} e_{0,t} & e_{1,t+\tau_{1}} & \cdots & e_{i,t+\tau_{1}+\cdots +\tau_{i}} & \cdots & e_{N,t+\tau_{1}+\cdots +\tau_{N}}\\ e_{0,t+T} & e_{1,t+\tau_{1}+T} & \cdots & e_{i,t+\tau_{1}+\cdots +\tau_{i}+T} & \cdots & e_{N,t+\tau_{1}+\cdots +\tau_{N}+T}\\ \vdots & \vdots & \ddots & \vdots & \ddots & \vdots \\ e_{0,t+(F-1)T} & e_{1,t+\tau_{1}+\left(F-1\right)T} & \cdots & e_{i,t+\tau_{1}+\cdots +\tau_{i}+\left(F-1\right)T} & \cdots & e_{N,t+\tau_{1}+\cdots +\tau_{N}+\left(F-1\right)T} \end{array}\right|$$

where $e_{0,*}$ and $e_{i,*}$ are the time series of the inlet process variables and the *i*th unit outlet process variables, respectively. $F\geq \sum_{1}^{N}d_{i}$, so that the data in the time-correlation matrix contain information about the entire process cycle, from inlet to outlet.

The matrix *E* describes the degree of correlation between the data corresponding to the time sequences. Multiple time sequences are described by the time-correlation analysis matrix,

(5) $$R=\frac{cov(E)}{\;\prod_{i=1}^{N}\sigma_{i}\;}$$

where cov(E) is the covariance matrix of *E*, whose standard deviation of column *i* is $\sigma_{i}$.

*R* is a time-correlation analysis matrix, which reflects the correlation degree of multiple groups of sequences under multi-delay. The $H_{\infty }$ norm can quantify it and is expressed as

(6) $$\beta =max(||R|{|}_{\infty })$$

A maximum value of the $H_{\infty }$ norm indicates the maximum correlation between each time sequence in the data matrix. At this time, the corresponding delay sequence Γ consists of the multi-delay to be solved.

According to the extraction production site experience, each unit’s time delay during operation will fluctuate within a fixed range. Based on this, the time-base value range of key process variables can be determined as $d_{i}\in \left[d_{imin},d_{imax}\right]$. Thus, the time delay identification problem of Equation (6) can be transformed into an unconstrained integer optimization problem,

(7) $$\left\{\begin{array}{c} \beta =\max({∥R∥}_{\infty })\\ s.t\;d_{i}\in \left[d_{imin},d_{imax}\right] \end{array}\right.$$

In summary, the steps of the improved method of combining grey correlation analysis with time-correlation analysis are as follows.

Step 1: Based on the original data of the rare earth extraction process, the multi-delay sequence Γ is constructed;

Step 2: Grey correlation analysis is used to identify key process variables and construct a time-correlation data matrix according to Equation (4);

Step 3: According to Equation (5), the time-correlation analysis matrix is defined, and the correlation degree of the data sequence is quantified by the $H_{\infty }$ norm;

Step 4: The time delay identification results are obtained using Equation (7).

There are many methods to solve the time delay identification. The discrete state transition algorithm (DSTA) has been successfully applied to typical discrete optimization problems such as Boolean integer programming [32] and staff assignment [30]. We use this method to solve Equation (7).

## 3. Adaptive Chaotic Discrete State Transition Algorithm

Discrete state transition algorithm is an individual-based optimization algorithm. Its basic idea is to regard the solution of an optimization problem as a state, and the process of updating the solution is called state transition. The standard form of the discrete state transition algorithm can be described as

(8) $$\left\{\begin{array}{c} x_{s+1}=A_{s}x_{s}⊕B_{s}u_{s}\\ y_{s+1}=f\left(x_{s+1}\right) \end{array}\right.$$

where $x_{s}\in Z$ is a current state; $A_{s}$, $B_{s}$ are transformation operators; $u_{s}\in Z$ is a control variable; ⊕ is an operation; f(·) is the evaluation function, which is used to measure the quality of $x_{s}$.

The four special transformation operators [32] are as follows.

(1) Swap transformation:
  (9) $$x_{s+1}=A_{s}^{swap}\left(m_{a}\right)x_{s}$$
  where $A_{s}^{swap}\in R^{n\times n}$ is a random 0–1 matrix with swap action, called a swap transformation matrix, and $m_{a}$ is a swap factor that can control the number of swap elements in the solution. Swap transformation is called local exploration and global exploration when $m_{a}=2$ and $m_{a}\geq 3$, respectively.
(2) Shift transformation:
  (10) $$x_{s+1}=A_{s}^{shift}\left(m_{b}\right)x_{s}$$
  where $A_{s}^{shift}\in R^{n\times n}$ is a random 0–1 matrix with shift action, called a shift transformation matrix, and a shift factor, $m_{b}$, can control the continuous shift of elements in the solution. If $m_{b}=1$, the shift transformation is regarded as local exploitation, and if $m_{b}\geq 2$, the shift transformation is regarded as global exploration.
(3) Symmetry transformation:
  (11) $$x_{s+1}=A_{s}^{sym}\left(m_{c}\right)x_{s}$$
  where $A_{s}^{sym}\in R^{n\times n}$ is a random 0–1 matrix with symmetry action, called a symmetry transformation matrix, and a symmetry factor, $m_{c}$, can control the continuous symmetry of elements in the solution. Symmetry transformation is intrinsically called global exploration.
(4) Substitute transformation:
  (12) $$x_{s+1}=A_{s}^{sub}\left(m_{d}\right)x_{s}+B_{s}^{sub}\left(m_{d}\right)u_{s}$$
  where $A_{s}^{sub}$, $B_{s}^{sub}\in R^{n\times n}$ is a substitute transformation matrix, and $m_{d}$ is a constant integer, called a substitute factor, to control the maximum number of positions to be substituted. If $m_{d}=1$, the substitute transformation is regarded as local exploitation, and if $m_{d}\geq 2$, the substitute transformation is regarded as global exploration.

The initial solution is given randomly in the discrete state transition algorithm, and the solution significantly affects the convergence performance of the algorithm. DSTA easily falls into local optima in the iterative process. Therefore, an adaptive chaotic discrete state transition algorithm (ACDSTA) is proposed by introducing the opposition-based learning strategy, chaotic perturbation strategy, and adaptive recovery strategy.

### 3.1. Initialization Method Based on Opposition-Based Learning Strategy

The initialization method of the discrete state transition algorithm has the problem of uneven distribution, which somewhat affects its optimization efficiency. Therefore, an opposition-based learning strategy (OBL) is introduced to initialize the discrete state transition algorithm. OBL [33] is a machine-learning method whose idea is to generate a reverse solution based on the forward solution, compare their fitness values, and select the optimal solution as the initial solution, thereby improving the optimization speed of the algorithm.

Let $X=[x_{1},x_{2},\ldots ,x_{c},\ldots ,x_{D}]$ be an entity in *D*-dimensional space. The reverse solution based on OBL is $X^{\prime }=\left[x_{1}^{\prime },x_{2}^{\prime },\ldots ,x_{c}^{\prime },\ldots ,x_{D}^{\prime }\right]$, where $x_{c},x_{c}^{\prime }\in \left[L_{a},L_{b}\right]$, c=1,2,…,D calculated as

(13) $$X^{\prime }=L_{a}+L_{b}-X$$

where $L_{a}$ and $L_{b}$ are the upper and lower bounds, respectively, of the value range of the target vector.

Based on the idea of OBL, the best initial solution is selected as

(14) $$y_{o}=\max\left(f\left(X\right),f\left(X^{\prime }\right)\right)$$

where f(·) is the fitness function, $y_{o}$ is the fitness value corresponding to $X_{o}$, and $X_{o}$ is the initial solution into the subsequent iterative process.

### 3.2. Chaos Perturbation Strategy

Chaos comes from nonlinear dynamic systems. Because of its unique randomness, ergodicity, and complexity, it can effectively prevent the algorithm from falling into local optima, and it is widely used in optimization problems [34]. We propose the chaotic perturbation strategy to improve the problem that the discrete state transition algorithm can easily fall into local optima and is described as follows. During the algorithm iteration, when a value recurs a certain number of times, it indicates that the algorithm has fallen into a local optimum. Chaotic perturbation is applied to obtain a chaotic variable sequence, which is inversely mapped to the original solution space to obtain the perturbation solution, which is substituted in the next iteration so that the calculation exits the local extremum. When the termination condition is satisfied, the algorithm ends the iteration, and the final solution is the global optimum.

There are many rules to generate chaos, among which logistic mapping is common, but it has the problem of uneven frequency distribution. Zhou et al. [35] combined logistic mapping and tent mapping based on the uniform ergodicity of tent mapping to realize logistic-tent mapping,

(15) $$X_{n+1}=\left\{\begin{array}{ccc} (\alpha X_{n}\left(1-X_{n}\right)+\left(4-\alpha \right)X_{n}/2)mod1 & \; & X_{n}<0.5\\ (\alpha X_{n}\left(1-X_{n}\right)+\left(4-\alpha \right)\left(1-X_{n}\right)/2)mod1 & \; & X_{n}\geq 0.5 \end{array}\right.$$

where $X_{n}\in \left[0,1\right]$ is a chaotic variable, and the mod1 operation ensures that its output data are in the range of [0,1], and α∈(0,4] is a chaotic factor, which we take as 3.99.

The chaotic variables generated by Equation (15) cannot be directly used for iterative calculation of the algorithm. So, chaotic variables are mapped to the solution space of the objective function,

(16) $$X_{new}=round\left(L_{b}+\left(L_{a}-L_{b}\right)X_{n}\right)$$

where $X_{new}$ is a new solution generated after chaotic perturbation.

### 3.3. Adaptive Recovery Strategy

In the iterative process of the discrete state transition algorithm, the chaos perturbation strategy is introduced to generate new solutions, which can effectively improve its ability to jump out of local optima. However, the new solution directly enters the next iteration, which will decrease the algorithm’s convergence performance. To only use the greedy criterion can no longer meet the convergence requirements. We propose adaptive recovery, adopting the greedy criterion to ensure the general convergence of the algorithm. The current value is restored to the preserved historical best value with adaptive probability to further improve convergence performance.

The discrete state transition algorithm is in the stage of rapid optimization in the early stages of iteration, which greatly decreases fitness. The recovery probability should be small at this time, so as not to affect the searchability of the algorithm in the early stage of iteration. In the middle and later stages, the searchability decreases, and the optimal historical value should be restored with a large probability. We use a nonlinear adaptive adjustment method [36],

(17) $$P=\left(p_{a}-p_{b}\right)\times \left(1-\sin\left(\frac{\pi }{2}\cdot {\left(\frac{iter}{iter_{max}}\right)}^{\mu }\right)\right)$$

where P∈[0,1], $P_{a}$ is the maximum value of the recovery probability *P*, $P_{b}$ is the minimum value of *P*, iter is the current number of iterations, $iter_{max}$ is the maximum number of iterations, and μ is the adaptive factor, which we take as 2.

The steps of the proposed adaptive chaotic discrete state transition algorithm are as follows.

Step 1: Set relevant parameters such as swap factor $m_{a}$, shift factor $m_{b}$, symmetry factor $m_{c}$, substitution factor $m_{d}$, chaos factor α, and adaptive factor μ;

Step 2: Generate the reverse solution using the initialization method according to Equation (13). The fitness of the forward and reverse solutions is compared by Equation (14) to select the best initial solution;

Step 3: Update the solution by swap, shift, symmetry, and substitution transformations (Equations (9)–(12)) in turn;

Step 4: According to Equation (17), judge whether the adaptive recovery strategy is satisfied, and if so, assign the optimal historical value to the current solution;

Step 5: Determine whether the condition of the chaotic perturbation strategy is satisfied. If so, generate the chaotic variable according to Equation (15), and the perturbation solution $X_{new}$ according to Equation (16) for the next iteration. Otherwise, go to step 6.

Step 6: Determine whether the iteration termination condition is satisfied. If so, terminate the search and output the final optimization result. Otherwise, return to step 3.

### 3.4. Validation of ACDSTA

Many applications in economics, chemistry, manufacturing, and other fields can be transformed into unconstrained integer optimization problems. To verify the feasibility, superiority, and applicability of ACDSTA, three functions of unconstrained integer optimization problems [37] are selected for experiments, denoted by EXP1, EXP2, and EXP3, and expressed as follows, with respective optimal values of −620, −70,429, and −1,439,658.

(18) $$\left[EXP1\right]\left\{\begin{array}{c} \min f\left(x\right)=\frac{1}{2}x^{T}Qx+c^{T}x\\ s.t\;x\in {\left\{0,1,2,\ldots ,10\right\}}^{8} \end{array}\right.$$

where

$$Q=\left(\begin{array}{cccccccc} 4 & -2 & -3 & 0 & 1 & 4 & 5 & -2\\ -2 & -4 & 0 & 0 & 2 & 2 & 0 & 0\\ -3 & 0 & 8 & -2 & 0 & 3 & 4 & 0\\ 0 & 0 & -2 & -4 & 4 & 4 & 0 & 1\\ 1 & 2 & 0 & 4 & 100 & 2 & 0 & -2\\ 4 & 2 & 3 & 4 & 2 & 100 & 1 & 0\\ 5 & 0 & 4 & 0 & 0 & 1 & 200 & 4\\ -3 & 0 & 0 & 1 & -2 & 0 & 4 & 10 \end{array}\right),$$

$$c^{T}=(-4\;\;\;1\;\;\;-8\;\;\;3\;-100\;-10\;-20\;\;0).$$

(19) $$\left[EXP2\right]\left\{\begin{array}{c} \min f\left(x\right)=x^{T}Qx\\ s.t\;x\in {\left\{0,1,2,\ldots ,49\right\}}^{10} \end{array}\right.$$

where

$$Q=\left(\begin{array}{cccccccccc} -1 & -2 & 2 & 8 & -5 & 1 & -4 & 0 & 0 & 8\\ -2 & 2 & 0 & -5 & 4 & -4 & -4 & -5 & 0 & -5\\ 2 & 0 & 2 & -3 & 7 & 0 & -3 & 7 & 5 & 0\\ 8 & -5 & -3 & -1 & -3 & -1 & 7 & 1 & 7 & 2\\ -5 & 4 & 7 & -3 & 1 & 0 & -4 & 2 & 4 & -2\\ 1 & -4 & 0 & -1 & 0 & 1 & 9 & 5 & 2 & 0\\ -4 & -4 & -3 & 7 & -4 & 9 & 3 & 1 & 2 & 0\\ 0 & -5 & 7 & 1 & 2 & 5 & 1 & 0 & -3 & -2\\ 0 & 0 & 5 & 7 & 4 & 2 & 2 & -3 & 2 & 3\\ 8 & -5 & 0 & 2 & -2 & 0 & 0 & -2 & 3 & 3 \end{array}\right).$$

(20) $$\left[EXP3\right]\left\{\begin{array}{c} \min f\left(x\right)=x^{T}Qx+c^{T}x\\ s.t\;x\in {\left\{0,1,2,\ldots ,99\right\}}^{20} \end{array}\right.$$

where

$$Q=\left(\begin{array}{cccccccccccccccccccc} -3 & 7 & 0 & -5 & 1 & 1 & 0 & 2 & -1 & -1 & -9 & 3 & 5 & 0 & 0 & 1 & 7 & -7 & -4 & -6\\ 7 & 0 & -5 & 1 & 1 & 0 & 2 & -1 & -1 & -9 & 3 & 5 & 0 & 0 & 1 & 7 & -7 & -4 & -6 & -3\\ 0 & -5 & 1 & 1 & 0 & 2 & -1 & -1 & -9 & 3 & 5 & 0 & 0 & 1 & 7 & -7 & -4 & -6 & -3 & 7\\ -5 & 1 & 1 & 0 & 2 & -1 & -1 & -9 & 3 & 5 & 0 & 0 & 1 & 7 & -7 & -4 & -6 & -3 & 7 & 0\\ 1 & 1 & 0 & 2 & -1 & -1 & -9 & 3 & 5 & 0 & 0 & 1 & 7 & -7 & -4 & -6 & -3 & 7 & 0 & -5\\ 1 & 0 & 2 & -1 & -1 & -9 & 3 & 5 & 0 & 0 & 1 & 7 & -7 & -4 & -6 & -3 & 7 & 0 & -5 & 1\\ 0 & 2 & -1 & -1 & -9 & 3 & 5 & 0 & 0 & 1 & 7 & -7 & -4 & -6 & -3 & 7 & 0 & -5 & 1 & 1\\ 2 & -1 & -1 & -9 & 3 & 5 & 0 & 0 & 1 & 7 & -7 & -4 & -6 & -3 & 7 & 0 & -5 & 1 & 1 & 0\\ -1 & -1 & -9 & 3 & 5 & 0 & 0 & 1 & 7 & -7 & -4 & -6 & -3 & 7 & 0 & -5 & 1 & 1 & 0 & 2\\ -1 & -9 & 3 & 5 & 0 & 0 & 1 & 7 & -7 & -4 & -6 & -3 & 7 & 0 & -5 & 1 & 1 & 0 & 2 & 1\\ -9 & 3 & 5 & 0 & 0 & 1 & 7 & -7 & -4 & -6 & -3 & 7 & 0 & -5 & 1 & 1 & 0 & 2 & 1 & 2\\ 3 & 5 & 0 & 0 & 1 & 7 & -7 & -4 & -6 & -3 & 7 & 0 & -5 & 1 & 1 & 0 & 2 & 1 & 2 & 3\\ 5 & 0 & 0 & 1 & 7 & -7 & -4 & -6 & -3 & 7 & 0 & -5 & 1 & 1 & 0 & 2 & 1 & 2 & 3 & 9\\ 0 & 0 & 1 & 7 & -7 & -4 & -6 & -3 & 7 & 0 & -5 & 1 & 1 & 0 & 2 & 1 & 2 & 3 & 9 & 4\\ 0 & 1 & 7 & -7 & -4 & -6 & -3 & 7 & 0 & -5 & 1 & 1 & 0 & 2 & 1 & 2 & 3 & 9 & 4 & -1\\ 1 & 7 & -7 & -4 & -6 & -3 & 7 & 0 & -5 & 1 & 1 & 0 & 2 & 1 & 2 & 3 & 9 & 4 & -1 & -3\\ 7 & -7 & -4 & -6 & -3 & 7 & 0 & -5 & 1 & 1 & 0 & 2 & 1 & 2 & 3 & 9 & 4 & -1 & -3 & 9\\ -7 & -4 & -6 & -3 & 7 & 0 & -5 & 1 & 1 & 0 & 2 & 1 & 2 & 3 & 9 & 4 & -1 & -3 & 9 & 7\\ -4 & -6 & -3 & 7 & 0 & -5 & 1 & 1 & 0 & 2 & 1 & 2 & 3 & 9 & 4 & -1 & -3 & 9 & 7 & -9\\ -6 & -3 & 7 & 0 & -5 & 1 & 1 & 0 & 2 & 1 & 2 & 3 & 9 & 4 & -1 & -3 & 9 & 7 & -9 & 8 \end{array}\right),$$

$$c^{T}=(-5\;2\;-1\;-3\;5\;4\;-1\;0\;9\;4\;7\;-4\;3\;5\;8\;-1\;1\;5\;-6\;9).$$

The algorithm was simulated using an Intel Core i5-11300H CPU at 3.10 GHz, with 16.00 GB RAM, Windows 10, and MATLAB R2018a, and compared with particle swarm optimization (PSO), DSTA, and DSTAI [32], based on the results of each group of experiments running 20 times.

The maximum numbers of iterations of EXP1–EXP3 were set to 100, 500, and 1000, respectively. The remaining parameters were set as follows: PSO learning probability $c_{1}=c_{2}=1.5$, initial population 120, inertia weight 0.8. The ACDSTA parameters were set to SE=30, $m_{a}=2$, $m_{b}=1$, $m_{c}=0$, $m_{d}=1$, and $m_{max}=20$. The parameter settings of DSTA and DSTAI were the same as in Zhou et al.’s study [32].

The average error, average value, and accuracy of optimization (S/20) were selected as performance evaluation indices of each algorithm. Table 1 compares the results of quadratic integer programming problems EXP1, EXP2, and EXP3, where the optimal values of each function index are in boldface.

**Figure 1:**
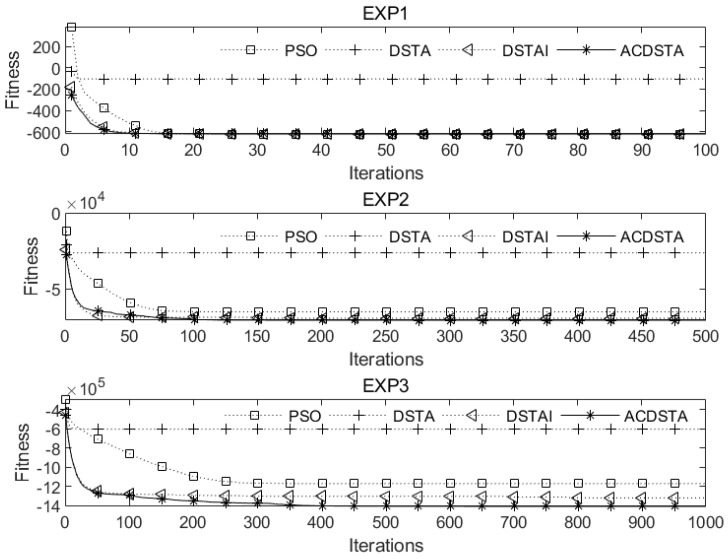
Convergencecurves of EXP1, EXP2, and EXP3 on four algorithms.

Figure 1 compares the convergence curves of EXP1, EXP2, and EXP3 under four optimization algorithms.

From Table 1 and Figure 1, we can see the following: (1) For the unconstrained integer optimization problem EXP1 with a low dimension, PSO, DSTAI, and ACDSTA can all find the optimal solution, and all the indices are the best; (2) For the unconstrained integer optimization problems EXP2 and EXP3 with higher dimensions, although PSO and DSTAI can also find the optimal solution, each index of ACDSTA is better. Among them, the optimization accuracies of EXP3 were improved by 65%, 75%, and 40% compared with PSO, DSTA, and DSTAI, respectively, and the respective average values were 20.3%, 133.1%, and 6.7% higher. Moreover, the average error was only 2.19%, which is significantly lower than for the other comparison algorithms. This indicates that the higher the dimensionality of the integer optimization problem and the larger the search space of the solution, the more prominent the advantage of ACDTSA; (3) Whether low-dimensional EXP1 or high-dimensional EXP3, ACDSTA was superior to the other three algorithms in terms of initial average fitness and convergence speed and could find the optimal value faster. This shows that ACDSTA can approach the optimal value faster and improve convergence performance through the opposition-based learning strategy and adaptive recovery strategy; (4) For EXP3, the optimization accuracy of ACDSTA was better than that of the other three algorithms. Although there was a tendency to fall into local optima in late iterations, it could effectively jump out of local optima and obtain better optimization accuracy because of the chaos perturbation strategy.

In summary, ACDSTA was superior to the other algorithms in the test of unconstrained integer optimization problems, especially when the dimension and optimization range were extensive, which can better reflect the advantages of ACDSTA. This demonstrates the feasibility, superiority, and applicability of ACDSTA when solving practical engineering problems.

## 4. Application of ACDSTA in Rare Earth Extraction Process

### 4.1. Rare Earth Extraction Process Analysis

**Figure 2:**
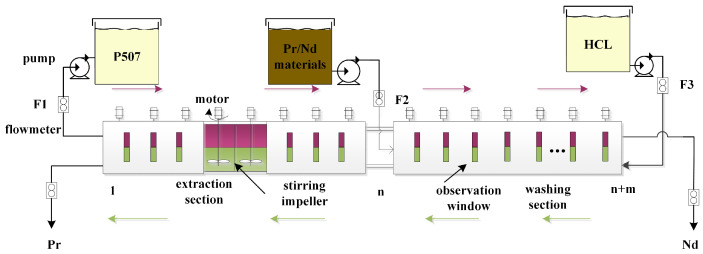
Schematic Diagram of Rare Earth Extraction Process.

Rare earth extraction is the obtaining of a single rare earth product from rare earth liquid, extractant, and detergent through specific equipment. We use the praseodymium/neodymium (Pr/Nd) extraction and separation process as an example, as shown in Figure 2.

The extractant is added from the first stage, and it flows from left to right through the stirrer. The detergent is added from the n+m stage and flows from right to left. The organic phase is added to the feed liquid from the nth stage. The solution in the tank is divided into two layers by stirring and clarifying. The upper layer is the organic phase, and the lower layer is the water phase. The difficult extraction product Pr is obtained in the lower layer of the first stage and the easy extraction product Nd from the upper layer of the n+m stage.

There are many extraction stages, and it is necessary to control the content distribution of Pr/Nd components in the tank to ensure the stability of product quality. It takes several hours or more to cause changes in the content of the export grade Pr/Nd component due to changes in the flow rate of the feed solution, extractant, and detergent. Therefore, it is necessary to obtain the residence time of materials in each unit group for timely control.

### 4.2. Time Delay Identification of Rare Earth Extraction Process

To verify the ability of the proposed method to solve the engineering delay problem, the delay identification of the 30-stage Pr/Nd production process in a rare earth extraction plant was carried out. During the Pr/Nd extraction process, the content of Pr/Nd components in different tanks changes with time, which leads to a change in the color of the solution. Therefore, the characteristic color variable of the solution image can be selected as an auxiliary variable to identify the time delay.

The sampling period was 5 min, and 220 groups of data of Nd component content and color characteristic variables (H, S, I, R, G, B) were selected in the continuous stable production process. The grey correlation method was used to analyze the correlation between Nd component content and color characteristic variables. The results are shown in Table 2, where the B component has the highest correlation degree, and the H component has the lowest correlation degree. Therefore, the B component is regarded as the critical process variable. In the actual extraction site, every five stages of the extraction tank share a set of agitators, i.e., every five stages constitute a unit group. Therefore, the 30-stage extraction tank was constructed as six groups of units for identification. According to the flow direction of the extractant, the first-stage inlet sampling data and each group of outlet sampling data were recorded as $e_{0},\ldots ,e_{6}$, and the original data matrix *E* was obtained.

According to the operation experience of the extraction separation site, the time delay range of stirring and clarification of each stage extraction tank is [3,8] min. Given the abovementioned construction method, the time delay range of each unit group is [15,40] min. Therefore, the value range of the time-base sequence is [3,8]. According to Equation (4), the time-correlation data matrix *E* is constructed, and the time delay sequence solution is transformed into the optimization problem by Equations (5) and (6), as shown in Equation (7).

**Figure 3:**
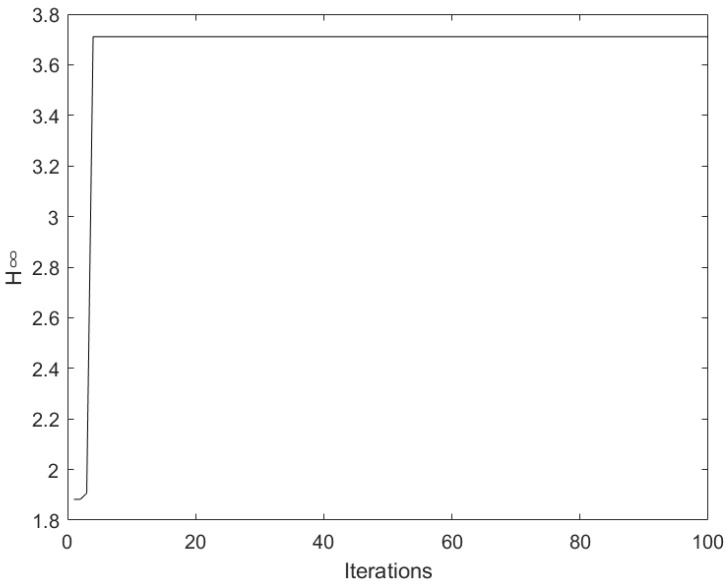
Iterative curve of improved discrete state transition algorithm.

ACDSTA is used to solve the abovementioned optimization problem. A certain number of time-based sequences is generated according to the range of time-based values, and the fitness function is constructed according to Equation (7). A new sequence is generated after the operation of the time-based sequence by the swap, shift, symmetry, and substitution operators. At the same time, the fitness value is calculated, and the current optimal value and the optimal time-based sequence are retained. The abovementioned operation is repeated until the iteration termination condition is satisfied, and the global optimal fitness value and global optimal time-based sequence are obtained. The solution process of ACDSTA is shown in Figure 3. Here, the maximum number of iterations is set to 100, SE=5, and the remaining parameters are set according to Section 3.4.

As seen from Figure 3, ACDSTA can obtain the optimal value $H_{\infty }=3.711$ in the early stage of iteration, and the corresponding time-base sequence is [364544]. Since the sampling period is 5 min, the time delay of the identified unit group is [153020252020], i.e., the 30-stage Pr/Nd extraction production process and the time delay identification result of each stage of the extraction tank are [333336666644444555554444444444].

### 4.3. Time Delay Identification Results and Method Verification

To verify the accuracy of the improved time delay identification method, we conducted the following experiments. Firstly, the characteristic components H, S, and I of the solution image of the rare earth extraction tank are considered auxiliary variables. A prediction model of Nd component content that meets the requirements of the extraction site is established by the wavelet neural network and used as a verification model. Then the maximum relative error (MAXRE), mean relative error (MEANRE), and mean absolute error (MAE) are determined to measure the performance of the model,

(21) $$MAXRE=MAX\left(\frac{\left|z-z^{\prime }\right|}{z^{\prime }}\times 100\%\right)$$

(22) $$MEANRE=\frac{1}{n}\sum_{i=1}^{n}\frac{\left|z-z^{\prime }\right|}{z^{\prime }}\times 100\%$$

(23) $$MAE=\frac{1}{n}\sum_{i=1}^{n}\left|z-z^{\prime }\right|$$

where *z* is the predicted component content value of the wavelet neural network, and $z^{\prime }$ is the actual component content value. Finally, the following comparative experiments are designed. Based on the data processed by the improved time delay identification method (Improved method), time-correlation analysis method (Original method), and Unused method, the performance of the component content model based on wavelet neural network (WNN) is compared and analyzed.

WNN is a neural network based on wavelet analysis theory that combines the excellent time-frequency localization property of wavelet function and the powerful self-learning function of the neural network. WNN uses the wavelet basis function as the activation function, which has a strong prediction ability than the back propagation neural network. Lu et al. [38] showed a nonlinear relationship between the color characteristic components H, S, and I of the rare earth extraction solution image and Nd component content. Therefore, we use the WNN to model the component content of the rare earth extraction process, using the Morlet function as the wavelet basis function.

(24) $$y=cos{\left(1.75x\right)}^{\left(-0.5x^{2}\right)}$$

**Figure 4:**
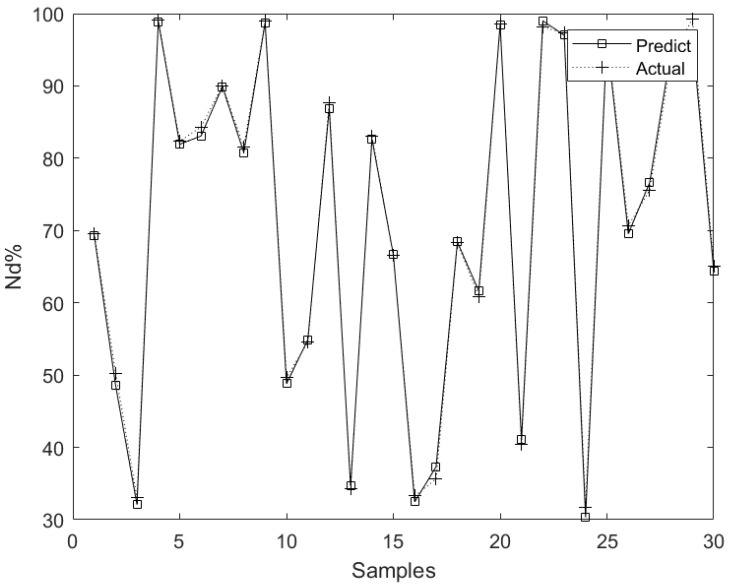
Prediction model result of wavelet neural network.

The parameter settings are as follows. The learning probability is 0.01, the momentum factor is 0.001, and the maximum number of iterations is 1000. The data samples consist of 220 groups, and 190 groups are randomly selected for model training. The remaining are used to verify the model, as shown in Figure 4.

The maximum relative error of the model prediction is 4.76%, which meets the accuracy requirements of the rare earth extraction site for the prediction model, so it can be used as a verification model for time delay identification.

**Figure 5:**
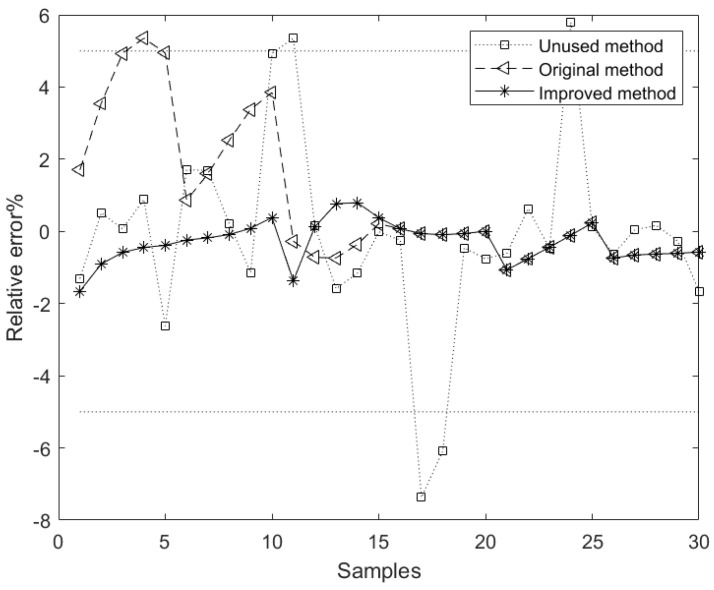
Forecast relative error of different methods.

Table 3 shows the model evaluation indices based on the Unused, Original, and Improved methods, where the bold data are the optimal values, and the corresponding relative error curve is shown in Figure 5.

From Table 3 and Figure 5, we can see the following: (1) The performance of the component content model based on the Original method is better than that based on the Unused method. However, its maximum relative error is greater than 5%, which does not meet actual requirements. This shows that although the Original method can improve the model’s performance to a certain extent, due to the randomness of its data selection and the lack of data preprocessing, the method fails to accurately identify the real-time delay. (2) The mean relative error of the component content model based on the Improved method is better than that based on the Unused and Original methods, which is reduced by 70.1% and 63.9%, respectively. This shows that the model based on the improved method is more stable. (3) Compared with the Unused and Original methods, the mean absolute error of the model based on the improved method is reduced by 60.6% and 58.6%, respectively, indicating that the prediction accuracy of the model is higher.

In summary, the improved method significantly improved the model’s performance. This shows that the improved method based on grey correlation analysis can effectively select the data closest to the real-time delay and improve the accuracy of the delay identification results. At the same time, the maximum relative error of the model based on the improved method is less than 5%, which meets the actual requirements. This shows that the improved time delay identification method proposed in this paper is suitable for the time delay identification of rare earth extraction process.

## 5. Conclusions

Rare earth extraction is a typical nonlinear, large-time-delay industrial process. The existence of time delay precludes the effective use of much field data and leads to a gap between the model describing the rare earth extraction process and the actual situation. We did the following work in response to this problem: Based on the standard discrete state transition algorithm, an improved algorithm (ACDSTA) was proposed, using an opposition-based learning strategy to initialize and accelerate the convergence of the algorithm, and an adaptive recovery strategy to improve its convergence performance. A chaotic perturbation strategy can improve the ability of the algorithm to jump out of local optima. An experimental comparison with three unconstrained integer optimization problems showed that ACDSTA can effectively solve such problems, and verified its effectiveness, superiority, and applicability; An improved time delay identification method was proposed to solve the problem that the data are not preprocessed and are randomly selected in the time-correlation analysis method. The method was applied to the time delay identification of a rare earth extraction process. The superiority and effectiveness of the proposed improved time delay identification method were verified by comparing the time-correlation analysis method and the data without the identification method under the same Nd component content. To sum up, the proposed time delay can provide a reference for modeling the rare earth extraction process and has guiding significance for the improvement of the online detection accuracy of component content.

## Figures and Tables

**Table 1:** Results of unconstrained integer optimization.

Algorithm	Index	EXP1	EXP2	EXP3
	average error	**0**	7.6%	18.7%
PSO	average value	**−620**	−64,907	−1,170,653
	S/20	**20/20**	11/20	2/20
	average error	82.9%	62.6%	58.1%
DSTA	average value	−106	−26,264	−603,975
	S/20	0/20	0/20	0/20
	average error	**0**	1.6%	8.4%
DSTAI	average value	**−620**	−69,148	−1,319,270
	S/20	**20/20**	18/20	7/20
	average error	**0**	**0**	**2.2%**
ACDSTA	average value	**−620**	**−70,429**	**−1,408,107**
	S/20	**20/20**	**20/20**	**15/20**

**Table 2:** Results of GRA.

Color Feature	R	G	B	H	S	I
correlation degree	0.6179	0.5832	**0.6734**	0.543	0.5706	0.6123

**Table 3:** Model evaluation indices of different methods.

Method	MAXRE%	MEANRE%	MAE
Unused	7.96	1.63	0.9183
Original	5.08	1.33	0.8738
Improved	**1.69**	**0.48**	**0.362**

## Data Availability

Not applicable.
